# Malignant pleural effusion: Updates in diagnosis, management and current challenges

**DOI:** 10.3389/fonc.2022.1053574

**Published:** 2022-11-17

**Authors:** Dinesh Narayan Addala, Nikolaos I. Kanellakis, Eihab O. Bedawi, Tao Dong, Najib M. Rahman

**Affiliations:** ^1^ Oxford Centre for Respiratory Medicine, Churchill Hospital, Oxford University Hospitals NHS Foundation Trust, Oxford, United Kingdom; ^2^ Oxford Pleural Unit, Oxford University Hospitals, Oxford, United Kingdom; ^3^ Oxford Biomedical Research Centre, National Institute for Health Research, Oxford, United Kingdom; ^4^ Nuffield Department of Medicine, Medical Sciences Division, Chinese Academy of Medical Science Oxford Institute, University of Oxford, Oxford, United Kingdom; ^5^ Department of Infection, Immunity and Cardiovascular Disease, University of Sheffield, Sheffield, United Kingdom; ^6^ Medical Research Council (MRC) Human Immunology Unit, Radcliffe Department of Medicine, Medical Research Council (MRC) Weatherall Institute of Molecular Medicine, University of Oxford, Oxford, United Kingdom

**Keywords:** pleural, oncology, malignant pleural effusion (MPE), thoracoscopy (pleuroscopy), indwelling pleural catheter (IPC)

## Abstract

Malignant pleural effusion (MPE) is a common condition which often causes significant symptoms to patients and costs to healthcare systems. Over the past decade, the management of MPE has progressed enormously with large scale, randomised trials answering key questions regarding optimal diagnostic strategies and effective management strategies. Despite a number of management options, including talc pleurodesis, indwelling pleural catheters and combinations of the two, treatment for MPE remains symptom directed and centered around drainage strategy. The future goals for providing improved care for patients lies in changing the treatment paradigm from a generic pathway to personalised care, based on probability of malignancy type and survival. This article reviews the current evidence base, new discoveries and future directions in the diagnosis and management of MPE.

## Introduction

Malignant pleural effusion (MPE) is the build-up of fluid between the lung and the chest wall as a result of cancer cells in the pleura. MPE is a common complication of cancer, with an incidence of 50 000 per year in the UK (1) and occurs in up to 15% of people with cancer. MPE can be associated with any type of cancer, both primary pleural malignancy (mesothelioma) and the result of secondary spread from other sites including lung, breast and ovarian (2). The effects upon patients living with MPE are profound, including significant breathlessness, fatigue and impact on daily activity (3). Furthermore, MPE is typically associated with poor prognosis and a median survival of 3-12 months (4). Recent data has indicated that the impact of MPE on healthcare is substantial, with the estimated annual national cost in the USA surpassing $1.5 billion and hospital readmissions leading to costs of $236 million annually (5). Over the past decade, the management of MPE has progressed significantly with an ever increasing number of high quality randomised trials to guide best practice (6–9). Despite the improving evidence base, a number of challenges persist in this vulnerable patient population including optimising time to diagnosis and definitive fluid control. The issue of survival in MPE is of great importance as it informs patient and physician decision making regarding management strategies. The importance of accurate survival scores is amplified in this cohort of patients, in whom balancing the short survival time with volume of hospital contact for fluid management is paramount. Nonetheless, prognostication has been amongst the more challenging aspects of MPE management, due to significant heterogeneity in both underlying malignancy and performance status of patients.

This article reviews the recent advances and standards of care in the management of MPE, while addressing the challenges and key areas requiring further targeted studies over the coming years to optimise the care of patients suffering with MPE.

## The current investigation and management of suspected malignant pleural effusion

**Figure 1:**
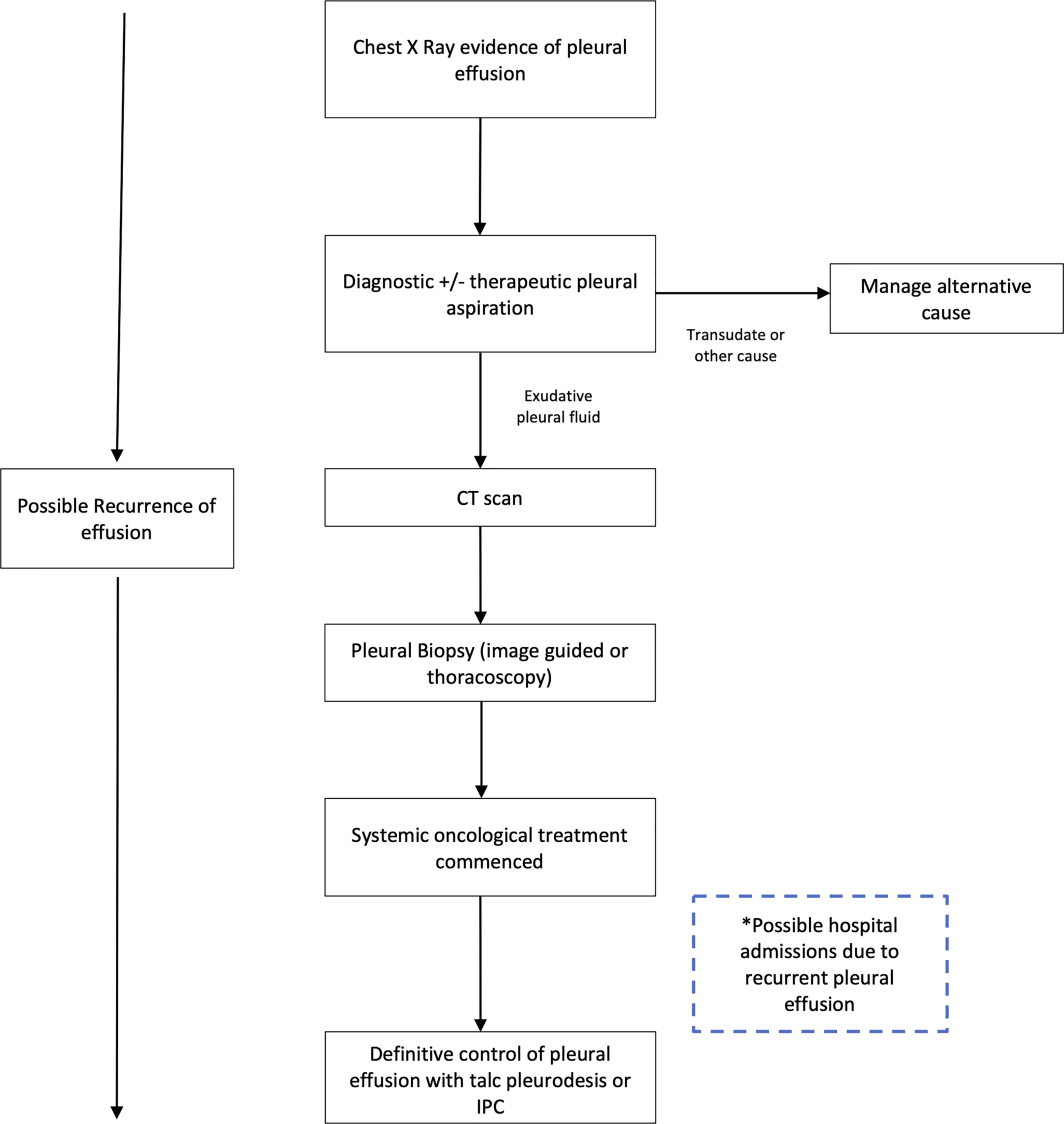
Current investigation and management pathway for diagnosis and management of malignant pleural effusion. Adapted from the British Thoracic Society Guidelines on management of pleural disease. CT, computed tomographic; IPC, Indwelling pleural catheter. Definitions: Transudate defined by pleural fluid with low protein and low lactate dehydrogenase (Light's criteria).

The current investigation and management pathway for a new pleural effusion is described in **Figure 1**

The pathway begins with a symptomatic patient presenting to either primary or secondary care with breathlessness, and basic imaging (chest radiograph) demonstrating a unilateral pleural effusion. The priorities for the patient and clinicians are to 1) establish a diagnosis while also 2) providing relief of symptoms. The initial procedure involves aspiration of pleural fluid with around 50mls sent for laboratory diagnostic analysis and assessment of cytology to establish a malignant diagnosis. In addition, a further 1-1.5 litres of fluid may be withdrawn to improve breathlessness.

Recent evidence suggests that the initial pleural aspiration may have limited utility in the diagnosis of MPE (10). The sensitivity of pleural fluid alone is low; even when malignant cells are detected, the sample may be insufficient to provide information to guide oncological treatment (10, 11) (‘actionable histology’), and the fluid recurs in the majority of patients. Following this first procedure, the patient therefore requires further procedures to achieve a diagnosis (pleural biopsy), and a further ‘definitive’ pleural fluid control procedure. This is conducted using either chemical pleurodesis to seal the pleural space or indwelling pleural catheter insertion (IPC) to control breathlessness and prevent re-admission to hospital. The relative merits and risks of these methods are evaluated below.

## Updates in diagnostics

### Imaging

In parallel to patient symptoms, an early indicator of pleural malignancy is imaging of the thorax. The most commonly utilised imaging modalities to assess potential MPE are chest radiograph, ultrasound and contrast-enhanced CT. Chest radiographs remain of utility as they are readily accessed from primary care and are often represent the most rapidly available diagnostic tool. Possible findings to indicate MPE include asymmetrical pleural effusions in the presence of either pleural thickening or a large lung mass (12). More subtle and detailed diagnostics however require either ultrasound or CT, as standard PA chest radiographs require approximately 200mls of pleural fluid for interpretation (13).

#### Ultrasound

Thoracic ultrasound (TUS) is now a significant part of the current standard of care for investigating MPE (14). TUS can detect smaller pleural effusions, alongside important predictors of malignant pleural disease causing pleural effusion such visceral and parietal pleural nodularity (15) as well as diaphragmatic nodules and thickening. The presence of pleural nodularity in conjunction with other features such as diaphragmatic nodularity or thickening on ultrasound has a positive predictive value for malignancy of between 83-100% (15, 16). Perhaps the most exciting paradigm shift for the use of ultrasound in MPE is it’s use as a dual diagnostic and therapeutic tool in MPE. Thoracic ultrasound forms a standard of care to guide pleural interventions providing increased safety and effectiveness compared with blind needle insertion (17). Recent data suggests that ultrasound can help identify non expansile lung (NEL) during pleural aspiration, and thus guide which patients may benefit from specific treatments, such as pleurodesis versus indwelling pleural catheter (18). Although these studies require further validation, the impact could be significant due to the poor sensitivity (24%) of chest radiograph to identify NEL, thus allowing earlier personalisation of treatment in MPE. The most robust data for the use of ultrasound as a therapeutic tool arises from the SIMPLE trial showing that a 9 point ultrasound scan of the thorax following talc pleurodesis can confirm pleural adherence to guide chest drain removal and reduce hospital stay by one day, while reducing health care costs (19).

#### Cross sectional imaging

Contrast CT imaging is an essential step in diagnosing MPE, providing a non-invasive modality to detect pleural features of malignancy such as circumferential pleural thickening, parietal pleural thickening > 1cm, and pleural nodules, with data suggesting these features carry a specificity of between 78-90% (20, 21). CT imaging is also required to assess for extra thoracic metastases and alternative sampling sites. CT is somewhat limited in cases where these features are absent, with a low negative predictive value for malignancy and thus further sampling of fluid or tissue is still required.

Some controversy exists around the utility of positron emission tomography (PET) in the diagnosis of pleural effusion caused by malignancy, with a modest reported specificity of 74%, and a sensitivity of 81% (22). Further confounders include the risk of false positives following talc pleurodesis or non-malignant inflammatory causes of pleural effusion. Thus PET scanning in the workup of MPE is not recommended routinely as part of international guidelines, however may have specific utility in providing biopsy targets for CT guided pleural biopsy where traditional investigations are precluded or have failed to secure a final tissue diagnosis (see below) (3, 23).

## Cytological vs. histological diagnosis – an evolving evidence base

### Pleural aspiration

For over a decade, international guidelines have advocated pleural aspiration as the first line investigation for suspected malignant pleural effusion. This typically involves withdrawal of sufficient pleural fluid for laboratory analysis and temporary relief of breathlessness. The diagnostic utility of pleural aspiration has, in recent studies, come into question. The diagnostic sensitivity of pleural fluid cytology is poor at only 37- 43% (11) of patients with proven MPE, and is worse with certain cancers (6% in mesothelioma). Repeat pleural fluid sampling has also been shown to add little to overall diagnostic rates (11). **Table 1** illustrates the rate of cytology positivity in specific cancer types. In addition, it is now clear that the finding of malignant cells in fluid alone is often insufficient to guide oncological treatment (24), with the increase in personalised oncological therapy requiring molecular markers to guide systemic therapy. As an example, in lung adenocarcinoma, current guidelines recommend assessment of multiple gene mutations prior to systemic treatment (25) with targeted treatment (such as immune modulating medication) offering the potential for favourable side effect profile and survival (26). In order to achieve this level of molecular marker analysis, tissue biopsy is often required, with fluid cytology likely to be insufficient. As a result, the case for a ‘direct to biopsy’ approach has been made (24) and future personalised strategies must target earlier biopsy when the cytological yield is most likely to be poor (for example in patients with a history of asbestos exposure and thus higher chance of mesothelioma).

**Table 1:** Pleural Fluid cytological sensitivity by cancer type, adapted from Arnold et al. (11).

	Pleural fluid sensitivity (%)
**All Cancer Types**	46.4 (42.0-58.2)
**Mesothelioma**	6.1 (2.8-11.2)
**Urological**	11.8 (1.5-36.4)
**Haematological**	40.0 (22.6-59.4)
**Lung**	56.0 (48.1-63.7)
Adenocarcinoma	82.0 (73.1-89.0)
Squamous	14.3 (4.0-32.7)
Small Cell	43.8 (19.8-70.1)
**Breast**	70.7 (57.3-81.9)
**Ovarian**	94.7 (82.2-99.4)

(95% confidence interval).

The therapeutic aspect of pleural aspiration, typically removing 1-2L of fluid from the pleural space alleviates symptoms due to an improvement in diaphragm function, and relief of the pressure effect on the diaphragm, rather than improvement in lung function (27). In the absence of symptomatic relief from therapeutic pleural aspiration, other common causes of breathlessness should be considered including pulmonary embolus or pneumonia. Although pleural fluid is prone to reaccumulating following pleural aspiration, the procedure does have utility in guiding the best strategy for definitive fluid control (e.g. with indwelling pleural catheter, IPC or chemical pleurodesis), by helping to identify non expansile lung (NEL). NEL occurs when pleural aspiration is associated with a negative pleural pressure resulting in chest pain. In pleural malignancy, entrapped lung due to visceral pleural thickening or endobronchial tumour, prevents complete lung re-expansion following drainage. In these cases, pleural drainage causes excessive negative pleural pressures (<20cm H20) leading to adverse symptoms. Pleural manometry has been used to measure pleural pressures during pleural drainage, and thus predict NEL, although using manometry does not appear to reduce the risk of procedural pain (28). Early identification of NEL is essential in informing patient discussions regarding definitive pleural fluid control – in patient’s whom the lung fully expands, viable options include chemical pleurodesis (which relies on pleural apposition) and IPC, whereas in those patients with NEL, IPC stands alone as the strategy of choice.

### Pleural biopsy

As noted above, in suspected MPE, histological analysis of pleural tissue obtained *via* biopsy is typically required to guide oncological treatment. The most commonly used pleural biopsy techniques include: ultrasound guided or CT guided pleural biopsy using a cutting needle visualised under image guidance, or thoracoscopic pleural biopsies, done under direct visualisation of the pleural space using a fibreoptic camera.

CT guided pleural biopsies have a similar diagnostic yield, providing adequate tissue for diagnosis in over 87% of patients and actionable molecular marker information in a high proportion (29). Ultrasound guided biopsies result in similar diagnostic yield (over 90%) however carry significant advantages pertaining to the patient pathway and waiting times. Ultrasound guided biopsies are typically faster to undertake, can be conducted by physicians at the first meeting with the patient without requiring CT scanners, and do not expose patients to ionizing radiation (30). Ultrasound guided biopsies can be performed by either physicians or radiologists, and can be combined easily with therapeutic drainage procedures such as IPC. CT guided biopsies require radiologists to undertake and are generally not combined with definitive fluid drainage. A key caveat that clinicians must bear in mind in regards to image guided biopsy techniques, is that the quoted diagnostic figures reflect instances where there is pleural nodularity or thickening identifiable with the imaging technique (an adequate ‘target’), and the diagnostic yield is likely to be significantly lower in the absence of this, in which case thoracoscopy should be performed. Recent data also indicates that the diagnostic sensitivity for molecular cancer markers is lower in image guided techniques than thoracoscopy (31).

Thoracoscopic biopsies are the preferred method of diagnosis for mesothelioma (3, 32), as larger tissue volumes are required and remain the overall gold standard diagnostic technique, with a diagnostic yield of 95% (33). Thoracoscopy facilitates direct visual inspection of the pleura and larger biopsies which are necessary to demonstrate fat or muscle invasion by tumour. Medical thoracoscopy can be performed under local anaesthetic and be combined with IPC insertion as a day case procedure by pleural physicians. The surgical alternative, video assisted thoracoscopic surgery (VATS) has a similar diagnostic yield to medical thoracoscopy however, carries increased risk of postoperative pain and requires general anaesthesia with single lung ventilation (34). Despite this, in selected cases, VATS is the preferred option, namely in those with significant pleural adhesions or septations that would preclude medical thoracoscopy, as these can be treated at the time of intervention in the case of VATS. Both medical thoracoscopy and VATS provide the opportunity to undertake therapeutic definitive fluid control measures by instilling a chemical sclerosant into the pleural cavity at the time of the procedure (35).

## Updates in the management of malignant pleural effusion

Recurrent pleural effusion caused by malignancy is often a debilitating condition for patients, with adverse effects on activity levels and performance status (36). The effects of MPE can also impact patients’ tolerance for systemic therapy resulting in a vicious cycle of pleural effusion build-up leading to lack of disease control measures such as missed systemic treatment. As such, patients and physicians are encouraged to openly discuss definitive management options for MPE, which despite great progress in recent years, remains palliative. The historical choice for prevention of pleural fluid recurrence has been chemical pleurodesis, with the greatest evidence base for graded talc as the agent of choice (37, 38). The chemical sclerosant precipitates a diffuse inflammatory reaction and fibrin deposition between parietal and visceral pleura, thus obliterating the pleural space and preventing fluid reaccumulation. The process typically requires an inpatient hospital stay for chest drain insertion and complete fluid drainage and is successful in approximately 70% of cases (6, 35). Although initially the success rate of pleurodesis was previously felt to be greater using talc poudrage at thoracoscopy, the TAPPS trial has shown pleurodesis failure rates at 90 days to be equivalent between poudrage and talc instillation *via* a chest tube (35).

IPCs are a silicone tube tunnelled under the subcutaneous tissue into the pleural space to allow ongoing drainage of pleural fluid in the patients home, by the patient themselves or a carer/nurse. IPCs carry some major advantages over talc pleurodesis, requiring only a day case procedure under local anaesthetic and providing equally effective management of MPE in patients with NEL. Over the past decade, IPCs have become the subject of a number of rigorous, high quality randomised clinical trials, with in depth evaluation of effectiveness in symptom control and cost effectiveness. Over this period these trials have not only seen a paradigm shift in the options offered to patients to manage MPE, but also the use of patient facing outcome measures (PROMS) as the primary outcome in the aforementioned clinical trials. These trials have shown that IPCs improve breathlessness and quality of life comparably to talc pleurodesis and reduce the length of hospitalisation by 2 days (6, 7). On this evidence alone, IPCs have become a viable option to patients with recurrent MPE, allowing a choice between IPC and talc according to patient preference. Whether IPCs should become a first line intervention *over* talc pleurodesis is however a more nuanced issue. IPC related complications occur in approximately 10-20% of patients, although the majority are minor and treatable, such as cellulitis, with true infection of the pleural space reported to be less than 5% (39, 40). In addition to this, the genuine impact of long term IPC drainage on both the patient and healthcare systems is significantly under-studied, as hospital admission days do not account for the limitations of requiring domiciliary drainage, often by a trained nurse. Even cost effectiveness studies do not clearly favour one intervention, with a *post hoc* analysis of the TIME-2 trial revealing that for patients with survival limited to <14 weeks, IPCs were more cost effective, however beyond this, talc pleurodesis was favourable (41).

Thus the choice between talc pleurodesis and IPC remains dependent on patient choice, resource availability and physician familiarity at the current juncture, although with the advent of combined treatments (see below), the balance of these factors may yet change. Spontaneous pleurodesis (autopleurodesis) has been reported with IPCs, and retrospective datasets have suggested the rate of autopleurodesis with IPCs to be 43-47% (37, 42), although in prospective studies suggest this is somewhat lower (24-27%) (9).

**Figure 2:**
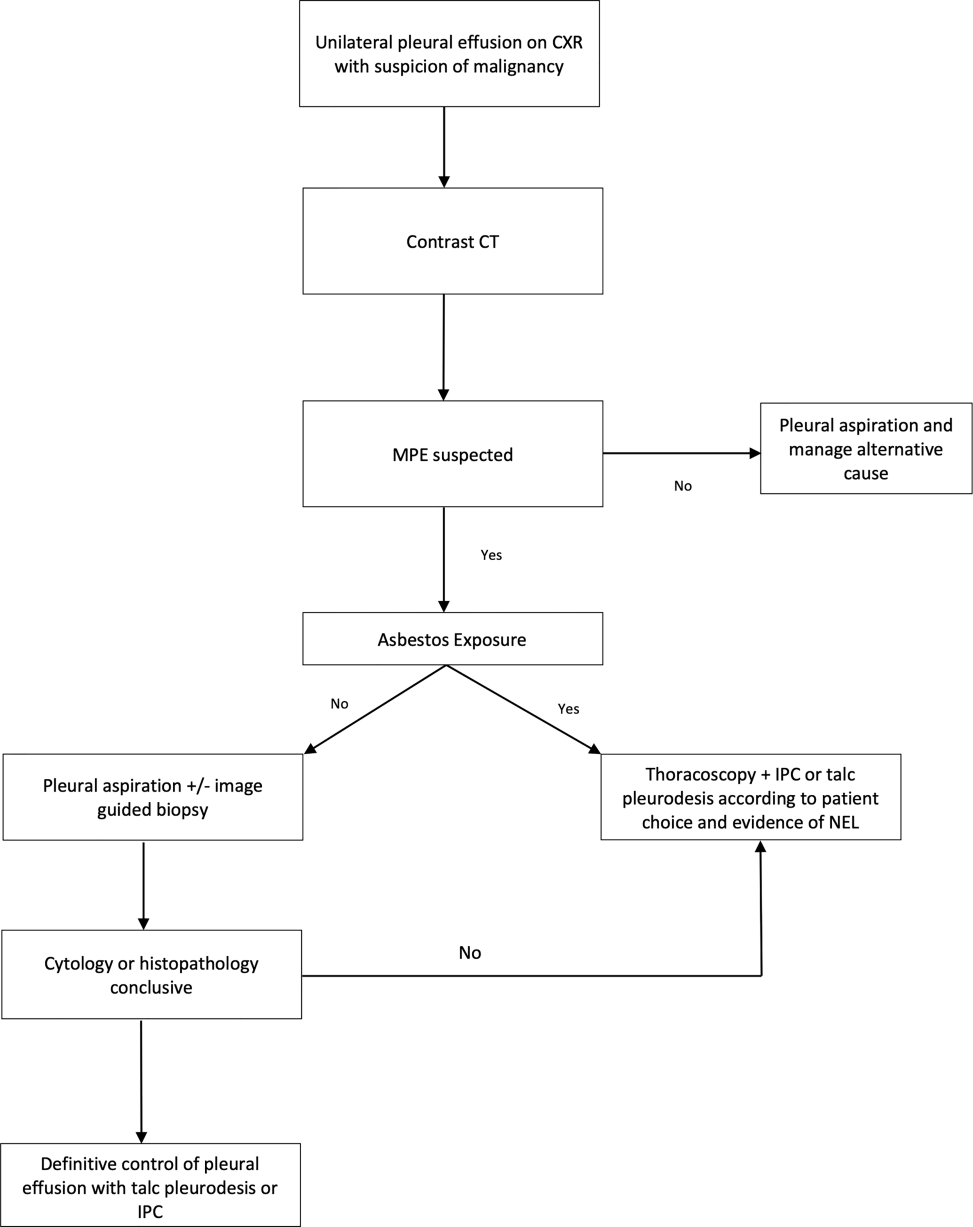
Authors suggested pathway for the investigation and management of suspected malignant pleural effusion based on current evidence. Diagnostic yield of tests noted: Pleural Aspiration 37-43% (11), Image guided pleural biopsy 84-93% (30), Thoracoscopy 95% (33). MPE, malignant pleural effusion; CT, computed tomography; IPC, indwelling pleural catheter; NEL, non expansile lung. Asbestos exposure determined by either imaging features such us pleural plaques or patient reporting. Asbestos exposure of importance due to increase in pre-test probability of mesothelioma.

**Figure 2** illustrates a suggested evidence based flowchart for the management of MPE, and the question future studies will seek to target is whether waiting times to definitive fluid control can be minimised or even delivered as part of a ‘first intervention’ for suspected MPE.

### Combination treatments

Recent large scale prospective studies have been undertaken to assess whether adjunctive treatments can improve the rate of pleurodesis with IPCs. The ASAP trial has shown that adopting an aggressive (daily) drainage strategy of IPCs over symptom guided drainage can almost double rates of autopleurodesis from 24% to 47% (9). The IPC PLUS randomised clinical trial showed that delivering talc as a sclerosing agent through an IPC can improve pleurodesis rates from 27% (standard care) to 51% at 70 days (8), showing promise for a combined treatment approach. It is essential to note however, that this outcome was in an enriched population wherein non expansile lung was excluded. This population is enriched as pleural apposition is required for successful pleurodesis, and typically not achieved in non expansile lung. If we compare this to the significantly higher rates of pleurodesis within the TAPPS trial *via* either talc poudrage or chest tube slurry (both with inpatient admission with no exclusion for NEL), it is clear that in cases where the patient’s priority is in achieving pleurodesis as a ‘one off intervention’, the optimal method is *via* poudrage or slurry.

The final technique that has been explored to improve pleurodesis rates *via* IPC is the use of a silver nitrate coated catheter, designed to initiate inflammation in the pleural space and encourage pleurodesis. While this had some initially promising results in animal studies and early trials (43, 44), a recent randomised trial of 119 patients (SWIFT), showed no improvement in pleurodesis efficacy with these devices compared to standard IPCs (45). As a result, no further studies of the silver nitrate coated catheter are currently in progress.

Potentially exciting developments in the decision making between inpatient and IPC based pleurodesis are on the horizon. The first of these is with the awaited results of the recently completed OPTIMUM randomised clinical trial (46). This novel trial used a quality of life based primary outcome to evaluate IPC plus talc versus standard talc slurry. The evaluation of these two modalities with a patient facing outcome may move the field further towards a clearer answer in this discussion. The second large scale study addressing this issue is the TACTIC trial (ISRCTN11058680), which is currently in recruitment, assessing the pleurodesis success rate of thoracoscopy with talc and inpatient admission versus thoracoscopy with talc and IPC insertion to allow ambulation.

## Prognostication and outcome prediction: The future of MPE management?

To date, prognostication in MPE has been addressed by two prognostic scoring systems validated in MPE; the LENT and PROMISE scores. The LENT score was derived using 3 prospectively collected datasets and retrospectively derived. LENT assigns patients to a low, moderate or high risk of death (319, 130, 44 days median survival respectively) using pleural fluid LDH, ECOG score, blood neutrophil-to-lymphocyte ratio (NLR), and tumour type (47). In a validation cohort, the LENT score was found to perform significantly better than ECOG performance status alone in predicting survival.

The PROMISE study stratified patients into four survival categories at three months ranging from <25% to >75%. The score includes clinical parameters (ECOG performance score, previous chemotherapy and radiotherapy, cancer type) and biological variables (white blood cell count, C-reactive protein, haemoglobin and serum levels of tissue inhibitor of metalloproteinases-1, TIMP-1) (48). Despite their simplicity (with the exception of TIMP-1 in PROMISE which is not routinely measured in clinical practice) and external validation, there has been suggestion that alternative scoring systems are necessary to correct for regional demographics variation, for example in areas with high rates of EGFR adenocarcinoma mutations (49) and target specific tumour types. As such one recent study has sought to address this using disease specific models to allow more precision in survival prediction. The breast and lung effusion survival score (BLESS) was derived retrospectively from analysis of 562 patients, and validated in a separate cohort of 727 patients. Both the lung and breast models utilise variables of ECOG performance status, benign pleural fluid cytology, pleural fluid LDH and pleural fluid protein. The lung model adds history of surgery within 30 days and the presence of bilateral pleural effusions. The breast model adds NLR. The authors concluded that in lung and breast malignancy, the BLESS score outperformed LENT, adding another potential tool to the prognostication of MPE (50). It remains to be seen whether these scoring systems become widely utilised in clinical practice however, as no studies to date have demonstrated clinical impact on patient reported outcomes or the clinical pathway in MPE.

An area with great potential to progress the management of patients with MPE is that of more sophisticated biomarker prediction of clinical outcomes including fluid volume prediction and autopleurodesis. Other fields have successfully integrated biomarker driven care pathways (51) and this remains lacking in MPE and pleural medicine in general. Important data has been discovered as part of the PROMISE study which aimed to discover and validate pleural fluid biomarkers to predict outcomes. Despite the analysis of over 1200 proteins, only 4 showed significant association with survival – TIMP-1, VCAN, GSN and MIF. Of note however, none of these could predict pleurodesis success, which for patients may represent a more direct impact on choice of fluid management strategy.

In regards to predictors of fluid output and autopleurodesis, early data suggests that routine clinical laboratory tests are not helpful in predicting outcome (42). A recent study has identified Vascular Endothelial Growth Factor (VEGF), Transforming Growth Factor-B (TGF-B) and Basic Fibroblast Growth Factor (FGF2) as key players in auto-pleurodesis induced by IPC (52). However, this was a longitudinal study and further studies are needed to assess these findings. A blind exploratory study to screen for auto-pleurodesis regulators has not been performed, which is a necessary next step to objectively identify the underlying molecular mechanisms underpinning autopleurodesis.

### Novel directions

Of great interest is early translational work showing that cancer cell cultures’ proliferation is promoted by seeding the cells in pleural fluid (53). This pro-growth property of pleural fluid opens up the possibility that pleural fluid may not be a bystander of malignant disease, requiring drainage only to provide palliation of symptoms, but may be an *active* promoter of cancer progression, thus emphasising the importance of achieving pleural fluid control early. Current strategies to do so are centred around mechanical methods of drainage and sealing the pleural space, however, in the coming years, significant efforts should be directed at more sophisticated biomarker analysis and validation and subsequent targeting of these with intrapleural immunological agents to ‘turn the tap off’. If successful, such treatment strategies have the potential to bring about a true stepwise change in the management of MPE. However significant challenges exist in this regard, in particular the clinical heterogeneity of MPE depending on primary tumour (54) and the evidence from studies of intrapleural treatments for MPE to date which have yielded mixed results (55, 56).

## Discussion

Over the last decade, great progress has been made in the treatment of MPE, with a formerly reactive and opportunistic approach moving to a robust evidence-based paradigm for both diagnostic and therapeutic options. Despite this, there remains key gaps in the evidence base. The true patient experience in MPE requires further assessment and the delivery of high yield diagnostics and definitive fluid control should be evaluated to determine if patients can progress to systemic treatment and symptom control earlier than current international guidelines allow. Over the next decade, moving beyond drainage strategies to biomarker and immunological analysis of MPE formation and recurrence will be essential, and may even lead to targeted pharmacological treatment of malignant pleural effusion.

## Author contributions

DA was responsible for initial draft preparation and revision. All other authors were responsible for reviewing and approving the final manuscript. All authors are responsible for the overall content.

## Conflict of interest

The authors declare that the research was conducted in the absence of any commercial or financial relationships that could be construed as a potential conflict of interest.

## Publisher’s note

All claims expressed in this article are solely those of the authors and do not necessarily represent those of their affiliated organizations, or those of the publisher, the editors and the reviewers. Any product that may be evaluated in this article, or claim that may be made by its manufacturer, is not guaranteed or endorsed by the publisher.
